# The Baby

**Published:** 1891-08

**Authors:** 


### THE BABY.

Nothing is more important to the health of a young infant than plenty of outdoor air—in the middle of the day in winter, early in the morning and in the evening in summer, and whenever it is pleasant and not too cold and damp or too sunny in autumn and spring. There are some very windy days in spring, when it is just as well, if the windy "spell" does not last more than a day, to keep the baby indoors. A windstorm is far more dangerous than rain. While no one would advise taking a baby out airing in a pelting rain, it is better to take him out in a slight snowstorm or "mere sprinkle" of rain than to keep him "cooped up" in the foul air of an unventilated nursery. The windows of the nursery or room where the child is kept should be open wide while it is out taking its airing, and closed and warmed up before it comes in. A slight fever which comes from teething or indigestion is often allayed by a walk in the pure open air. This is nature's own medicine, more soothing than all the sleeping draughts in the world to a restless, nervous child. In the summer, so far as practicable, a baby should live outdoors under the apple trees, or in any dry place not exposed glaringly to the rays of the sun. A nap taken in his carriage under the apple blossoms will be more soothing than one in the nursery within doors.

Bathing should be begun from the day of birth. Let no foolish old wife's stories cause you to omit it. A young infant is apt to cry at first, but this is as good exercise as he can have at the time, always provided it is not a severe screaming. Bathing is a necessity to perfect health, in the development of infants. The fact that certain children, like swine, seem to thrive in neglect and dirt, is only a proof of the vigor of their physical nature, which will exist in apparent health under such unwholesome surroundings. Such children often develop afterwards scrofulous sweatings. It has been repeatedly proved in tenement districts of large cities that only a comparatively few of the children, and those the more vigorous, born in squalor and dirt, survive. When a child is restless at night, a warm bath—about 90 degrees is the right temperature—with a cup of rock-salt dissolved in it, is wonderfully soothing. Only a pure soap should be used. A little fine starch is the best baby powder. It is hardly safe to use any other powder about a young child.

